# The Histone Demethylase Inhibitor GSK-J4 Is a Therapeutic Target for the Kidney Fibrosis of Diabetic Kidney Disease via DKK1 Modulation

**DOI:** 10.3390/ijms23169407

**Published:** 2022-08-20

**Authors:** Peir-Haur Hung, Yung-Chien Hsu, Tsung-Hsien Chen, Cheng Ho, Chun-Liang Lin

**Affiliations:** 1 Department of Internal Medicine, Ditmanson Medical Foundation Chia-Yi Christian Hospital, Chiayi 600566, Taiwan; 2 Department of Applied Life Science and Health, Chia-Nan University of Pharmacy and Science, Tainan 717301, Taiwan; 3 Department of Nephrology, Chang Gung Memorial Hospital, Chiayi 613016, Taiwan; 4 Kidney and Diabetic Complications Research Team (KDCRT), Chang Gung Memorial Hospital, Chiayi 613016, Taiwan; 5 Division of Endocrinology and Metabolism, Chang Gung Memorial Hospital, Chiayi 613016, Taiwan; 6 School of Traditional Chinese Medicine, College of Medicine, Chang Gung University, Taoyuan 333423, Taiwan; 7 Kidney Research Center, Chang Gung Memorial Hospital, Taoyuan 333423, Taiwan; 8 Center for Shockwave Medicine and Tissue Engineering, Chang Gung Memorial Hospital, Kaohsiung 833253, Taiwan

**Keywords:** diabetic kidney disease, streptozotocin-induced diabetic mice, histone demethylase, fibrosis, inflammation, DKK1

## Abstract

Diabetic kidney disease (DKD) can cause inflammation and fibrosis, in addition to being the main complication of diabetes. Among many factors, epigenetic alterations in aberrant histone modifications play a key role in causing DKD. In this study, the mechanism of GSK-J4, a histone demethylase KDM6A inhibitor, was evaluated in streptozotocin-induced diabetic mice. It was confirmed that GSK-J4, via dickkopf-1 (DKK1) modulation, could significantly reduce proteinuria and glomerulosclerosis in diabetic mice. The mRNA accumulation levels of DKK1, TGF-β1, fibronectin, and collagen IV were significantly elevated in diabetic mice. In contrast, the mRNA accumulations of those genes were significantly reduced in diabetic mice treated with GSK-J4 compared to those in diabetic mice, relatively speaking. The protein accumulation levels of fibronectin and collagen IV were significantly elevated in diabetic mice. Furthermore, GSK-J4 attenuated the high glucose-induced expression of profibrotic factors in mesangial cells via DKK1. In conclusion, our study provides a novel strategy to eliminate fibrosis in the kidneys of DKD mice. Using GSK-J4 reduces DKK1 expression, thereby ameliorating renal insufficiency, glomerulosclerosis morphological abnormalities, inflammation, and fibrosis in diabetic mice.

## 1. Introduction

Diabetic kidney disease (DKD) leads to chronic kidney disease (CKD) and end-stage renal disease (ESRD), which is a microvascular complication in patients with diabetes that results in the chronic loss of renal function [1,2]. DKD has high morbidity and mortality in patients with diabetes mellitus (DM) [3]. Diabetic patients are known to develop DKD due to genetic and environmental factors, and early lesions of DKD include the accumulation of extracellular matrix (ECM), hypertrophy, and glomerular as well as interstitium fibrosis [4,5]. One of the main features of DKD is chronic renal inflammation and fibrosis, in which inflammatory cytokines stimulate tubular epithelial cells to secrete collagen I and fibronectin. Additionally, inflammatory cytokines also lead to interstitial ECM deposition and basement membrane thickening, accelerating tubulointerstitial fibrosis [1].

Dickkopf-1 (DKK1) is a Wnt antagonist that antagonizes Wnt signaling by triggering the internalization of coreceptors LRP5/6, and it is also a high-affinity ligand of Kremen proteins [6]; it is also involved in other signaling pathways, including the c-Jun NH2-terminal kinase (JNK) pathway and DKK1/CKAP4 pathway. The JNK pathway is implicated in diabetes [7], inflammatory bowel disease [8], cardiovascular disease [9], cancer [10,11], and others, where the loss of JNK signaling results in the inhibition of tumor necrosis factor (TNF)-stimulated cell death [12]. DKK1 responds to the JNK signaling cascade, which increases DKK1 expression when the cellular sensitivity to various stresses activates JNK signaling [13]. Furthermore, DKK1 acts through the DKK1/CKAP4/PI3K pathway and increases the expression of plasma-membrane-vesicle-associated proteins, which is positively correlated with angiogenesis in cancer cells [14]. Hyperglycemia increases the mesangial cell expression of DKK1, the Kremen-2 receptor, transforming growth factor-β (TGF-β), and fibrotic factors [15], thus ultimately escalating to damage to the glomerular filtration barrier, leading to DKD. TGF-β also activates Wnt signaling, while Wnt/β-catenin signaling regulates TGF-β-mediated cell fibrosis [16]. TGF-β stimulates Wnt signaling via reducing the Wnt expression antagonist DKK1 [16]. DKK1 reduces β-catenin phosphorylation and attenuates TGF-β1 expression, thereby preventing fibrosis [15]. Furthermore, TGF-β-mediated histone modifications [17] are associated with the progression of DKD [18,19,20].

Epigenetic alterations in abnormal DNA methylation or histone modifications also play a key role in causing renal injury [1]. Histone methylation tightens the chromatin structure and inhibits the binding of transcription factors to promoters [21]; the extent of histone methylation can regulate gene transcription [22] and affects the pathogenesis of DKD [23]. For example, methylation at histone H3 lysine 27 (H3K27) is associated with gene repression. The loss of the H3K27me3 mark in podocytes also leads to podocyte dedifferentiation and glomerular damage through the inactivation or deletion of H3K27 methylase [24]. Conversely, the pharmacological inhibition of demethylating enzyme KDM6B, which specifically demethylates H3K27 trimethylation, attenuated glomerular disease [24]. The aim of this study was to investigate the functional role of GSK-J4, an inhibitor of H3K27 demethylase, in renal fibrosis, using a streptozotocin (STZ)-induced murine model of diabetes mellitus and renal fibrosis. Our results suggest that GSK-J4 is an important epigenetic regulator that effectively reduces renal fibrosis and dysfunction during renal injury.

## 2. Results

### 2.1. GSK-J4 Significantly Reduced Proteinuria in STZ-Induced Mice

After C57BL/6 mice were treated with STZ, we found a significant increase in blood glucose and serum hemoglobin A1c (HbA1c), as well as in the ratio of the kidney to body weight, total urine protein to creatinine, and albumin to creatinine (Table 1). Moreover, the kidney/body weight ratios in the STZ-induced diabetic mice increased, but the body weight decreased (*p* < 0.05).

Following treatment with GSK-J4, we found that STZ-induced diabetic mice saw a decrease in proteinuria (urine protein and albumin) (*p* < 0.05). The kidney/body weight ratios decreased significantly in the STZ-induced diabetic mice where GSK-J4 was administrated (*p* < 0.05). However, no significant difference in the blood glucose and HbA1c levels were found in the mice that were only STZ-induced or STZ-induced with the administration of GSK-J4.

### 2.2. GSK-J4 Reduced Glomerular Collagen Deposition in STZ-Induced Mice

**Figure 1:**
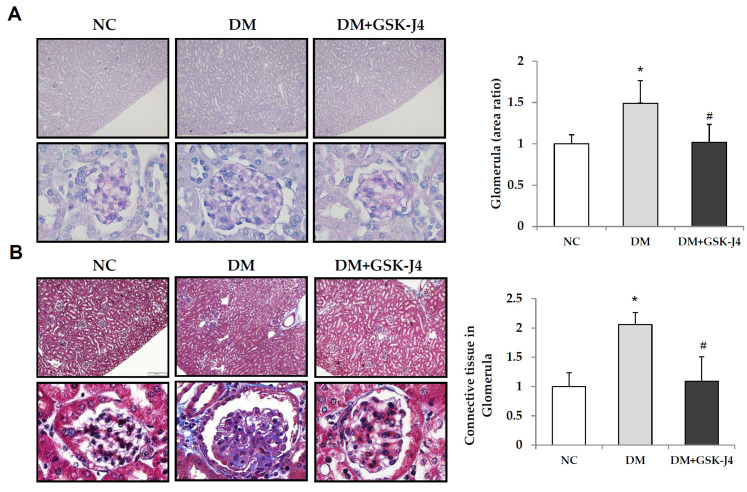
Periodic acid–Schiff (PAS) staining and Masson trichrome staining of kidneys from the mice. The mice were divided into three groups: normal mice (NC), STZ-induced diabetic mice (DM), and STZ-induced diabetic mice with GSK-J4 treatment (DM + GSK-J4). (**A**) Kidneys were stained with a PAS stain to detect glomerular damage. Glycogen, acid, and neutral epithelial mucin are stained magenta; nuclei are stained blue. (**B**) Masson trichrome staining of fibrosis in kidneys. The connective tissue is stained blue, nuclei are stained dark red/purple, and the cytoplasm is stained red/pink. (Enlargement factor: upper, 100×; lower, 1000×.) The right panel shows relative quantification of the glomerular area and connective tissue in the glomerular. * *p* < 0.05 versus the control group; ^#^ *p* < 0.05 DM versus DM + GSK-J4. Abbreviations: DM, STZ-induced diabetic mice; NC, normal mice.

In histopathological changes in the periodic acid–Schiff (PAS) staining, we observed an increase in the area and accumulation of glycogen staining in the glomerulus of the STZ-induced diabetic mice. Typical features of renal changes in DKD can be observed, including glomerular hypertrophy, glomerular basement membrane thickening, mesangial dilatation, and tubular dilation. In contrast, the administration of GSK-J4 significantly attenuated histopathological lesions in the kidneys of the STZ-induced diabetic mice (Figure 1A). In addition, the staining areas of collagen and fibrin were observed in excessive accumulation in the glomeruli of the STZ-induced diabetic mice with Masson staining, which show overexpressed collagen and fibrin. However, the administration of GSK-J4 significantly reduced the staining areas of collagen and fibrin accumulation in the glomerulus of STZ-induced diabetic mice (Figure 1B).

### 2.3. GSK-J4 Reduced DKK1 and TGF-β1 in STZ-Induced Mice

**Figure 2:**
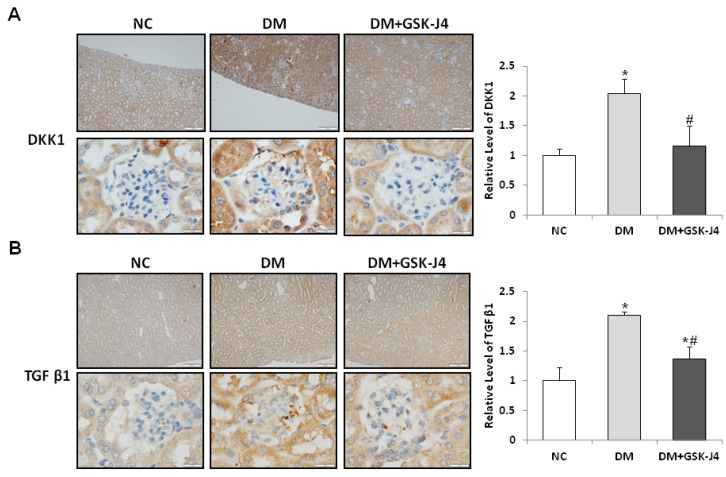
GSK-J4 reduces DKK1 and TGF-β1 expression in the glomeruli of STZ-induced diabetic mice. The mice were divided into three groups: normal mice (NC), STZ-induced diabetic mice (DM), and STZ-induced diabetic mice with GSK-J4 treatment (DM + GSK-J4). Immunohistochemistry detection for related proteins of DKK1 (**A**), and TGF-β1 (**B**). The right panel shows the relative quantification of DKK1 and TGF-β1 in the tissue immunostaining analysis. The average ratio of the integrated optical density (IOD) to the area (IOD/pixel) was used to analyze the results of immunohistochemistry. The statistical results were compared with the normal control (NC) group. * *p* < 0.05 versus the control group; ^#^ *p* < 0.05 DM versus DM + GSK-J4. Data are mean± standard errors calculated from six mice. Abbreviations: DKK1, dickkopf-1; DM, STZ-induced diabetic mice; NC, normal mice; and TGF-β1, transforming growth factor β1. (Enlargement factor: upper, 100×; lower, 1000×).

In the immunohistochemical sections for DKK1 and TGF-β1, the positive staining areas were limited to a small scale when GSK-J4 was administered, whereas the accumulation of DKK1 and TGF-β1 was relatively high in the STZ-induced diabetic mice (Figure 2). Semiquantification revealed the accumulation of DKK1 and TGF-β1 in the STZ-induced diabetic mice. The protein-labeled regions of DKK1 and TGF-β1 were both increased in the glomeruli of the STZ-induced diabetic mice, while in the STZ-induced diabetic mice that were administered GSK-J4, the protein-labeled areas of DKK1 and TGF-β1 were relatively reduced.

### 2.4. GSK-J4 Reduced Glomerulosclerosis in STZ-Induced Mice

**Figure 3:**
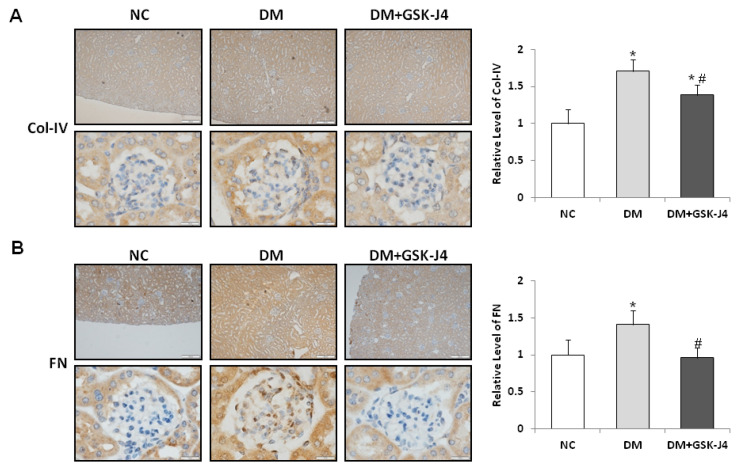
GSK-J4 reduces fibrosis in the glomeruli of STZ-induced diabetic mice. The mice were divided into three groups: normal mice (NC), STZ-induced diabetic mice (DM), and STZ-induced diabetic mice that were treated with GSK-J4 (DM + GSK-J4). Immunohistochemistry detection for fibrosis biomarkers revealed proteins of (**A**) collagen IV (Col-IV) and (**B**) fibronectin (Fn). The right panel shows the relative quantification of fibrosis biomarkers in the tissue immunostaining analysis. The average ratio of the integrated optical density (IOD) to the area (IOD/pixel) was used to analyze the results of immunohistochemistry. The statistical results were compared with the normal control (NC) group. * *p* < 0.05 versus the control group; ^#^ *p* < 0.05 DM versus DM + GSK-J4. Data are mean ± standard errors calculated from six mice. Abbreviations: DM, STZ-induced diabetic mice; NC, normal mice (enlargement factor: upper, 100×; lower, 1000×).

**Figure 4:**
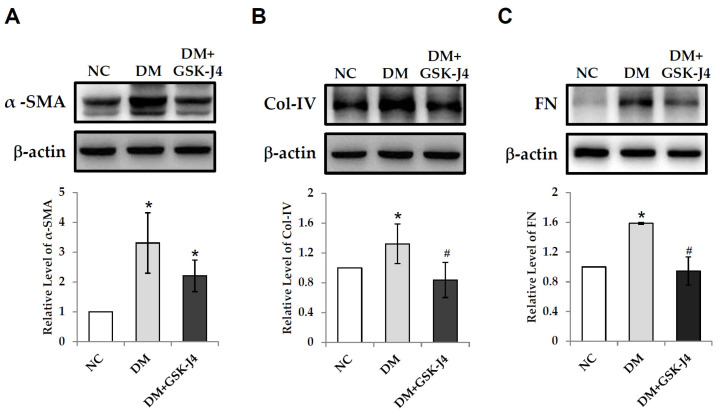
GSK-J4 reduces renal fibrosis-associated proteins in STZ-induced diabetic mice. Western blot analysis of kidney protein levels in C57BL/6 mice (NC), STZ-induced diabetic mice (DM), and STZ-induced diabetic mice that were treated with GSK-J4 (DM + GSK-J4) that were detected using (**A**) α-SMA, (**B**) collagen IV, and (**C**) fibronectin antibodies. The lower panel shows the relative quantification of α-SMA, collagen IV, and fibronectin in the Western blot analysis, respectively. * *p* < 0.05 versus the control group; ^#^ *p* < 0.05 DM versus DM + GSK-J4. Abbreviations: DM, STZ-induced diabetic mice; FN, fibronectin; Col-IV, collagen IV; and NC, normal mice.

Fibrosis-related proteins, fibronectin, and collagen IV were further analyzed to determine the effect of the administration of GSK-J4 on antifibrotic activity. In STZ-induced diabetic mice, immunostained sections showed increased staining areas in which fibronectin and fibrosis-associated proteins of collagen IV were identified (Figure 3). There was a significant increase in the amount of α-SMA, fibronectin, and fibronectin as well as collagen IV protein accumulations (Figure 4), whereas in STZ-induced diabetic mice that were treated with GSK-J4, the staining areas of fibronectin and collagen IV fibrosis-related proteins were reduced relative to those that were not treated with GSK-J4 (Figure 3). In addition, the accumulation of α-SMA, fibronectin, and collagen IV proteins in the kidneys of the diabetic mice that were given GSK-J4 was also significantly lower than in those that were not given GSK-J4 (Figure 4).

### 2.5. GSK-J4 Downregulates the Glomerular mRNA Expression of DKK1, TGF-β1, Fibronectin, and Collagen IV in Diabetic Mice

**Figure 5:**
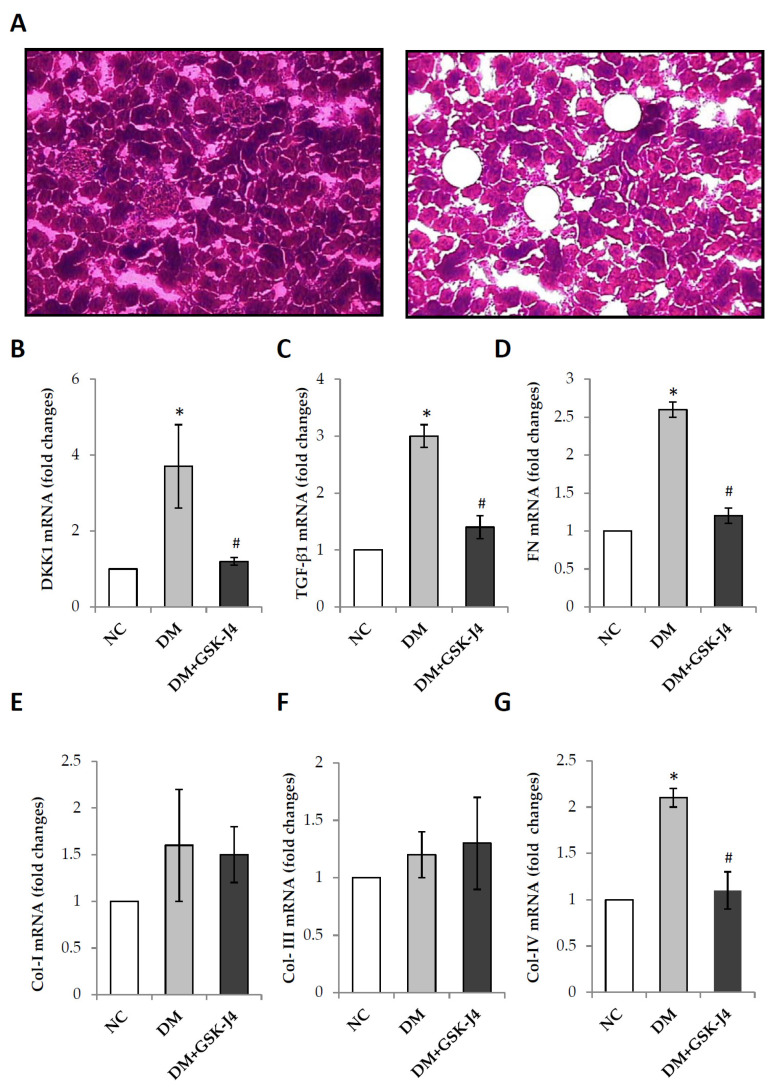
The mRNA expression levels of profibrotic genes in the glomerular of STZ-induced diabetic mice after laser capture microdissection (LCM). For the experiment, the mice were divided into three groups: (i) normal mice (NC), (ii) STZ-induced diabetic mice (DM), and (iii) STZ-induced diabetic mice that were treated with GSK-J4 (DM + GSK-J4). (**A**) Laser capture microdissection of glomeruli in formalin-fixed paraffin-embedded kidney tissues. Before LCM (**left**); after LCM (**right**). These samples were then separately collected for RNA and subjected to quantitative RT-PCR analysis for DKK1 (enlargement factor 10×). (**B**), TGF-β1 (**C**), FN (**D**), Col-I (**E**), Col-III (**F**), and Col-IV (**G**). * *p* < 0.05 versus the control group; ^#^ *p* < 0.05 DM versus DM + GSK-J4. Data are mean ± standard errors calculated from six mice. Abbreviations: Col-I, collagen I; Col-III, collagen III; Col-IV, Collagen IV; DKK1, dickkopf-1; DM, STZ-induced diabetic mice; FN, fibronectin; NC, normal mice; and TGF-β1, transforming growth factor β1.

Glomerular sections of tissue sections were collected with laser capture microdissection, and the total RNA was extracted for a subsequent quantitative RT-PCR analysis. The relative mRNA expression of DKK1, TGF-β1, fibronectin, and collagen IV was higher in STZ-induced diabetic mice as compared with the controls. The relative mRNA expression of DKK1 was higher in STZ-induced diabetic mice as compared with those that were administered GSK-J4 (3.7 ± 1.1 vs. 1.2 ± 0.1, *p* < 0.05). The relative mRNA expression of TGF-β1 was higher in STZ-induced diabetic mice as compared with those that were administered GSK-J4 (3.0 ± 0.2 vs. 1.4 ± 0.2, *p* < 0.05). The relative mRNA expression of fibronectin was higher in STZ-induced diabetic mice as compared with those that were administered GSK-J4 (2.6 ± 0.1 vs. 1.2 ± 0.1, *p* < 0.05). Furthermore, the relative mRNA expression of collagen IV was higher in STZ-induced diabetic mice as compared with those that were administered GSK-J4 (2.1 ± 0.1 vs. 1.1 ± 0.2, *p* < 0.05). Furthermore, the relative mRNAs of collagen I and collagen III were not significantly different in STZ-induced diabetic mice compared with normal mice or those that were administered GSK-J4 (1.6 ± 0.6 vs. 1 and 1.5 ± 0.3; 1.2 ± 0.2 vs. 1 and 1.3 ± 0.4, respectively) (Figure 5).

### 2.6. GSK-J4 Attenuates HG-Induced Profibrotic Factor Expression in Mesangial Cells via DKK1

**Figure 6:**
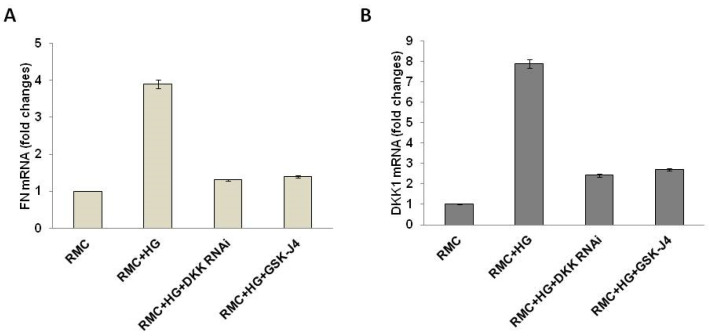
GSK-J4 attenuates high glucose-induced profibrotic factor expression in mesangial cells via DKK1. Cells were divided into four groups: rat renal mesangial cells (RMCs) were cultured in a basal medium containing glucose for 48 h (RMC + HD), after which DKK-1 RNAi (RMC + HD + DKK RNAi) or GSK-J4 (RMC + HD + GSK-J4) was added. Quantitative RT–PCR analyses of (**A**) FN and (**B**) DKK1. Abbreviations: FN, fibronectin; DKK1, dickkopf-1; HG, high glucose; and RMCs, rat kidney mesangial cells.

High concentrations of glucose increased the relative mRNA expression of DKK1 and fibronectin in renal mesangial cells (7.9 ± 0.21, 3.9 ± 0.11) as compared with the control. DKK1 siRNA significantly attenuated the relative mRNA expression of DKK1 and fibronectin in glucose-induced cells (2.42 ± 0.21 vs. 7.9 ± 0.21, 1.3 ± 0.02 vs. 3.9 ± 0.11) as compared with the glucose-induced cells, respectively. In addition, GSK-J4 also significantly attenuated the relative mRNA expression of DKK1 and fibronectin in glucose-induced cells (2.71 ± 0.06 vs. 7.9 ± 0.21, 1.40 ± 0.03 vs. 3.9 ± 0.11) as compared with the glucose-induced cells, respectively (Figure 6).

## 3. Discussion

The H3K27 demethylase inhibitor ameliorated the early diabetic kidney disease lesions in diabetic mice [25]. The inactivation or deletion of methylase leads to the loss of the H3K27me3 mark in podocytes resulting in podocyte dedifferentiation and glomerular damage. Additionally, the imbalanced expression of DKK1 and nuclear β-catenin promotes hyperglycemia-mediated cell apoptosis and the synthesis of the fibrotic matrix in DKD [15,26]. However, the mechanism of action of DKK1 in renal fibrosis remains questionable. A previous study demonstrated that KDM6A, specifically, demethylated H3K27me2, plays a pivotal role in promoting DKD, as well as the fact that the GSK-J4 inactivation of KDM6A enzymatic activity in diabetic mice led to decreased KDM6A levels [27]. In this study, we extend these findings to further demonstrate the role of GSK-J4 in regulating diabetes-induced glomerulosclerosis via DKK1.

The pathological and clinical features of DKD are glomerular basement membrane thickening, mesangial cell aggregation, renal fibrosis, and proteinuria, as well as multiple mechanisms known to be involved [1]. Multiple signaling pathways are involved in the pathological process of nephritis and renal fibrosis during DKD. For example, the Wnt signaling pathway is involved in the progression of DKD [28] and a variety of cellular behaviors, such as cell proliferation, migration, adhesion, and polarity [29]. Wnt/β-catenin regulates TGF-β1-mediated mesangial cell fibrosis and activates glycogen synthase kinase-3β (GSK-3β) signaling [30], thereby inducing mesangial cell fibrosis and apoptosis [26]. In addition, the Smad signaling pathway mediated by TGF-β1 is a representative signaling pathway leading to DKD. The TGF-β1/Smad3 signaling pathway mediates renal fibrosis and inflammation in addition to promoting the development of DKD [31].

The main features of DKD include mesangial fibrosis, etc. The TGF-β1 signaling pathway promotes the activation of fibroblasts and the abnormal synthesis of the fibrotic matrix in mesangial cells [32]. TGF-β1 promotes cell proliferation and differentiation, extracellular matrix synthesis [33], and tubular endothelial-mesenchymal transition (EndMT), leading to the development of tubulointerstitial fibrosis [34,35]. TGF-β1 induced the hypermethylation of the Rasal1 (Ras–Gap-like protein 1) promoter and decreased RASAL1 gene expression, thereby increasing fibroblast activation and fibrosis, resulting in renal fibrosis [36]. TGF-β1 is abundantly expressed in kidney and infiltrating inflammatory cells, and regulates many signaling pathways, including Smad signaling pathways [37]. Additionally, TGF-β1 signaling is involved in mediating profibrotic responses through non-Smad pathways [37].

The overexpression of Wnt and the downregulation of DKK1 were found in human fibrotic disease samples, whereas increased DKK1 expression significantly attenuated TGF-β1-induced fibrosis in animal models [16]. DKK1 inhibits Wnt signaling, which results in decreased β-catenin phosphorylation and attenuated TGF-β1 expression, leading to reduced fibrosis in mesangial cells [15]. DKK1 inhibits fibroblast-specific protein 1, collagen I, and fibronectin, which subsequently reduces renal fibrosis in obstructed kidneys [38]. Under diabetic conditions, KLF10 induces KDM6A expression, causing proteinuria and irreversible kidney damage [27]. DKK1 also attenuated nuclear localization and increased the phosphorylation of β-catenin [15]. The depletion of KLF10 suppresses DKK1 and TGF-β1, and phosphorylated β-catenin expression ameliorates diabetic renal fibrosis. Additionally, Hus et al. found that miR-29a regulates the DKK1/Wnt/β-catenin signaling pathway and TGF-β1-mediated renal tissue fibrosis, in addition to maintaining Wnt/β-catenin signaling activity to prevent diabetes-induced extracellular matrix accumulation and renal injury [39].

Histone acetylation can relax the chromatin structure and promote the binding of transcription factors to promoters, thereby enhancing transcriptional activation. The critical role of histone was acetylation in renal fibrosis. The miR-29 was accompanied by the increased expression of nephrin, which is known to reduce histone deacetylase (HDAC) 4 expression and result in the elevated expression of nephrin [40]. HDAC 4 is required for TGF-β1-induced myofibroblastic differentiation. The inhibition of histone deacetylation or the silencing of HDAC 4 expression inhibits α-SMA gene transcription [41]. In addition, Chen et al. demonstrated that the administration of GSK-J4 ameliorated diabetes-induced renal abnormalities in db/db (BKS.Cg-Dock7m^+/+^ Leprdb/JNju) mice, an animal model of type 2 diabetes [25]. In this study, the administration of GSK-J4 in STZ-induced mice also ameliorated diabetes-induced renal abnormalities. Thus, the administration of a histone demethylase inhibitor improves renal abnormalities in animal models of congenital and acquired diabetes. However, the efficacy of the administration of histone demethylase inhibitors to prevent renal abnormalities in animal models of acquired diabetes is unknown. Thus, more research is needed.

## 4. Materials and Methods

### 4.1. Animals

Mice were maintained in standard pathogen-free facilities in the Laboratory Animal Center, Department of Medical Research, Chang Gung Memorial Hospital at Chiayi, Taiwan. All mice were housed with a 12-hour light/dark cycle during the maintenance process, with a constant temperature (20–25 °C) and humidity (40–60%). All of the animal experiments were approved by the Institutional Animal Care and Use Committee of the Chang Gung Memorial Hospital (No. 2015061902).

### 4.2. Diabetic Mice Model Induction and GSK-J4 Treatment Protocol

Diabetes mellitus was induced in 12-week-old male C57BL/6 mice (BioLasco Biotechnology Co., Taiwan) through an intraperitoneal injection of STZ (200 mg/kg body weight) according to the standard method [42]. Before injection, the STZ (Merck, St. Louis, MO, USA) was dissolved in a sodium citrate buffer (pH 4.5) to a final concentration of 20 mg/mL. After the intraperitoneal injection of STZ into the C57BL/6 mice, normal chow and water of 10% sucrose were also provided. On day 3, the 10% sucrose water was replaced with regular water. On day 10, the mice fasted for 6 h, then, tail vein blood samples were collected to measure the blood glucose. Mice were analyzed for three consecutive analog fasting glucose concentrations at 200–300 mg/dL, confirming that the mice were diabetic, as previously reported [15].

This experiment was divided into three experimental groups, with six mice in each group; Group 1: normal mice; Group 2: STZ-induced diabetic mice; and Group 3: STZ-induced diabetic mice that were treated with GSK-J4. GSK-J4 (0.4 mg/kg, 4594; Tocris Bioscience, Bristol, UK) was administered subcutaneously daily for one week. In the fifth week after the experimental treatment, the kidney tissue of each mouse was collected for subsequent analysis.

### 4.3. Urine and Blood Biochemistry

Each mouse’s urine excretion was collected at the bottom of the metabolic cage in measuring cylinders. The method steps for measuring the total protein in urine were as previously described [40]. According to the method of the corresponding kits, the levels of HbA1c (BioVision, Milpitas, CA, USA) and blood glucose (Crystal Chem, Elk Grove Village, IL, USA) were measured.

### 4.4. Quantification of Glomerulosclerosis

The mice’s kidney tissues were fixed in 4% paraformaldehyde and then embedded in paraffin. The tissue was sliced off the 5-micron-thick kidney sections for staining by the PAS (Sigma-Aldrich Inc., St. Louis, MO, USA) and Masson’s trichrome (HT-15; SigmaAldrich) staining methods. The glomerular capillary loops and tubular epithelium, highlighting basement membranes, can be visualized by PAS staining. Masson’s trichrome staining was used to examine glomerular collagen deposition (blue). The areas of positive immunolabeled regions were quantified by using NIH ImageJ software. The average ratio of integrated optical density (IOD) to the area (IOD/pixel) was used to analyze the results of immunohistochemistry. PAS staining directly selects the glomerular area by the manual circle, while Masson’s trichrome staining first uses the color deconvolution, then uses the manual circle to select the glomerular area, and then quantifies. The statistical results were compared with the normal control group.

### 4.5. Immunohistochemistry

The kidney tissue slices were deparaffinized with xylene and rehydrated by graded alcohol. The antigen retrieval was performed for 15 min in the Tris-EDTA PH 9.0 in the microwave oven. After blocking the samples with 3% H_2_O_2_ for 10 min, the slides were incubated with Blocking Reagent for 60 min.

DKK1 (1:250 dilution; sc-25516; Santa Cruz, Dallas, TX, USA), TGF-β1 (1:250 dilution BS1361; Bioworld Tech, St Louis Park, MN, USA), fibronectin (1:250 dilution, F2372; Bioworld Tech, St Louis Park, MN, USA), and collagen IV (1:250 dilution, Abcam, Trumpington, Cambridge, UK) antibodies were used to identify specific proteins. Horseradish peroxidase-3′-, 3′-diaminobenzidine kits (R & D Systems, Minneapolis, MN, USA) were used for dyeing. Five glomeruli in each section were randomly selected for microscopy under 100× magnifications (Carl Zeiss, Gottingen, Germany). The quantitative analysis of positive immunolabeled regions was performed using NIH ImageJ software. The average ratio of the integrated optical density (IOD) to the area (IOD/pixel) was used to analyze the results of immunohistochemistry. For immunohistochemical staining, glomerular regions were directly selected by manual circle selection and then quantified. The statistical results were compared with the normal control group.

### 4.6. Laser Capture Microdissection

Glomerular portions are harvested using laser capture microdissection technology, as has been previously described [43]. Renal tissues were covered with laser capture microdissection transfer film (CapSure TF-100; Arcturus Engineering, Inc., Mountain View, CA, USA). The specific glomerular portions of the tissue section, and those which were under the RNAase-free conditions, were obtained using a VERITAS™ laser-captured dissection system (Arcturus Bioscience Inc., Sunnyvale, CA, USA), as described previously [15]. Each group collected 200 glomeruli from 6 sections per mouse for quantitative RT-PCR analysis.

### 4.7. Quantitative Reverse Transcription-PCR (RT-PCR)

Methods for total RNA extraction, the total RNA treated with DNase I, cDNA preparation, and quantitative PCR have been described previously [15,44]. Specific primers of quantitative RT-PCR analysis for detecting DKK1, TGF-β1, fibronectin, collagen IV, and β-actin were obtained from Ambion Inc. (Austin, TX, USA) (5′-TCCGTCTGCCTCCGATCATC-3′ and 5′-GCCTTTCCGTTTGTGCTTGG-3′ for DKK1; 5′-TGAGTGGCTGTCTTTTGACG-3′ and 5′-TGGGACTGATCCCATTGATT-3′ for TGF-β1; 5′-GACCCCGAGGTTAGGAAGG-3′ and 5′-CACTCGGTCCATGATCCCA-3′ for collagen IV; 5′-GTGGCTGCCTTCAACTTCTC-3′ and 5′-AGTCCTTTAGGGCGGTCAAT-3′ for fibronectin; and 5′-CGCCAACCGCGAGAAGAT-3′ and 5′-CGTCACCGGAGTCCATCA-3′ for β-actin). Relative quantification fold change was performed using the ΔCt method, with the formula 2^−ΔΔCt^, where ΔΔCt = ΔCt_treatment_ − ΔCt_vehicle_ and ΔCt = Ct_target_ − Ct_β-actin_. Each experimental group had at least six independent replicates.

### 4.8. Western Blot of Kidney Tissue

Protein was collected from the kidney using a cell lysis reagent plus a protease inhibitor (Roche, Basel, Schweiz). SDS-PAGE separated proteins in total kidney lysates (40 ug), which were then analyzed by Western blot. The designated proteins on the blots were probed with mAbs against α-SMA (1:1000 dilution, Abcam, Trumpington, Cambridge, UK), fibronectin (1:1000 dilution, Bioworld Tech, Nanjing, China), collagen IV (1:1000 dilution, Novus Biologicals, Littleton, CO, USA), and actin (Cell Signaling, Danvers, MA, USA), followed by horseradish peroxidase–conjugated IgG as the secondary antibody (1:5000, Jackson ImmunoResearch Laboratories, West Grove, PA, USA), and visualized by enhanced chemiluminescence (Merck, Darmstadt, Germany).

The quantification of the Western blot was carried out according to the NIH ImageJ software (1.53k; National Institutes of Health; Bethesda, MD, USA) standard analysis method. The rectangular box (box tool) was used to select the entire band to be quantified. The signal peaks in the rectangular box were quantified, and the peak area was calculated as the IOD value of each band.

### 4.9. In Vitro Cell Model Using DKK-1 RNAi and GSKJ4

Rat kidney mesangial cells (RMC Line, American Type Culture Collection, Manassas, VA, USA) were maintained in DMEM containing 10% FBS (Life Technologies, Carlsbad, CA, USA) in 5% CO_2_ at 37 °C, and then harvested by trypsinization for subsequent studies. RMCs were cultured in a basal medium containing 35 mM D-glucose for 48 h.

The pLKO-1-puro plasmids encoding DKK-1 RNAi (TRCN0000055154), which targeted DKK-1 mRNA, were obtained from the National RNAi Core Facility Academia (Sinica, Taiwan). Subconfluent cell cultures were transiently transfected with DKK1 siRNA (1 μg) and scramble controls using Lipofectamine 2000 (Invitrogen, Waltham, MA, USA), according to the manufacturer’s instructions. In the other group, glucose-induced cells were treated with 10 mM GSKJ4.

### 4.10. Statistical Analyses

The data are expressed as the mean ± standard errors. Student’s *t*-test was used to evaluate differences between each group. *p* < 0.05 was considered statistically significant.

## 5. Conclusions

This is the first study to characterize the beneficial effects of GSK-J4’s downregulation of DKK1 on the kidney. DKK1 effectively decreased diabetes-induced glomerular fibrosis. GSK J4 attenuates fibronectin and collagen IV expression in STZ-induced glomerular cell fibrosis via the decreased expression of DKK1 and TGF-β1.

## Figures and Tables

**Table 1:** Biochemical properties.

	Control (B6)	DM (B6 + STZ)	DM (B6 + STZ) + GSK-J4
Blood glucose (mg/dL)	149 ± 13	491 ± 47 *	483 ± 62 *
HbA1c (%)	3.9 ± 0.2	9.2 ± 1.2 *	9.0 ± 2.1 *
Body weight(g)	31.2 ± 0.9	24.9 ± 0.8 *	24.1 ± 1.2 *
Kidney/body weight (%)	0.81 ± 0.07	1.27 ± 0.19 *	0.94 ± 0.03 *^,#^
Urine (Tp/Cr) (mg/day)	6.1 ± 0.2	9.0 ± 1.1 *	5.9 ± 0.6 ^#^
Urine (albumin/Cr) (μg/day)	7.1 ± 0.3	53.4 ± 1.9 *	41.9 ± 3.1 *^,^^#^

* *p* < 0.05 versus control group; ^#^ *p* < 0.05 DM versus DM + GSK-J4. Data are mean ± standard errors calculated from six mice. Cr, creatinine; DM, STZ-induced diabetic mice; HbA1c, serum hemoglobin A1c; STZ, streptozotocin; and TP, urine protein.

## Data Availability

The data presented in this study are available on request from the corresponding author.
